# Alterations in the default mode network in rolandic epilepsy with mild spike-wave index in non-rapid eye movement sleep

**DOI:** 10.3389/fnins.2022.944391

**Published:** 2022-08-09

**Authors:** Yihan Li, Yingfan Wang, Ping Jiang, Jintao Sun, Qiqi Chen, Xiaoshan Wang

**Affiliations:** ^1^Department of Neurology, The Affiliated Brain Hospital of Nanjing Medical University, Nanjing, China; ^2^MEG Center, Nanjing Brain Hospital, Nanjing, China

**Keywords:** rolandic epilepsy, default mode network, magnetoencephalography, spectral power, functional connectivity

## Abstract

**Purpose:**

Rolandic epilepsy (RE) is one of the most common epilepsy syndromes during childhood. The aim of this study was to investigate the alterations in the default mode network (DMN) of RE patients whose spike-wave index (SWI) was within the 50–85% range during non-rapid eye movement (NREM) during sleep, as well as to detect early neuroimaging markers.

**Methods:**

Resting-state data was recorded for each subject using magnetoencephalography (MEG). DMN-related brain regions were chosen as regions of interest. The spectral power and functional connectivity (FC) of the DMN were estimated through the use of minimum norm estimation (MNE) combined with Welch technique and corrected amplitude envelope correlation (AEC-c).

**Results:**

The patient group included 20 patients with NREM phase 50% ≤ SWI < 85% (mild SWI group), and 18 typical RE patients (SWI < 50% group). At the regional level, the mild SWI group exhibited enhanced spectral power in the delta band of the bilateral posterial cingulate cortex and attenuated the spectral power in the alpha band of the bilateral posterial cingulate cortex. Enhanced spectral power in the bilateral precuneus (PCu) in the delta band and attenuated spectral power in the right lateral temporal cortex (LTC) in the alpha band were common across all RE patients. At the FC level, patients in the mild SWI group indicated increased AEC-c values between the bilateral posterial cingulate cortex in the delta band and between the left medial frontal cortex (MFC) and bilateral posterial cingulate cortex in the alpha band. Increased AEC-c values between the right PCu and left MFC in the delta band, and between the left PCu and right MFC in the theta band, were common across all RE patients. Moreover, the spectral power in the bilateral posterial cingulate cortex in the alpha band and the AEC-c value between the bilateral posterial cingulate cortex in the delta band demonstrated good discrimination ability.

**Conclusion:**

The spectral power of the bilateral posterior cingulate cortex (PCC) in the alpha band and the AEC-c value between the bilateral PCC in the delta band may be promising indicators of early differentiation between mild SWI and typical RE.

## Introduction

Rolandic epilepsy (RE), also known as benign epilepsy with central temporal spikes (BECTS), is one of the most common epilepsy syndromes during childhood (Drury and Beydoun, 1991). The disease develops most often between the ages of 6 and 13 years, and has a significant age-dependence (Wirrell, 1998). Most patients experience partial seizures, seizures that are closely related to sleep and occur only during sleep in about 75% of children with the disease (Wirrell, 1998). During the interictal period, the electroencephalogram (EEG) background activity has been shown to be normal, and single or clustered spike or spike waves are seen in the central and temporal regions on one or both sides (Wirrell, 1998). The abnormal discharges are found to be closely related to sleep, and are significantly increased during sleep (Clemens and Majoros, 1987).

The previous belief was that the course of RE was self-limiting and that the prognosis was always good. Furthermore, there was no residual neuropsychological damage (Blom and Heijbel, 1982). With advances in neuropsychological testing, as well as the development of brain imaging techniques, more and more scholars have proposed that RE is a non-benign disorder and that some RE patients exhibit atypical symptoms and clinical test results (Filippini et al., 2015; Bektaş et al., 2019; Curnow et al., 2020). For example, abnormal EEG features, different from typical RE, having repeated failures of conventional antiseizure medications (ASMs) therapy, and poorer neuropsychological cognitive impairment (Fejerman, 2009; Garcia-Ramos et al., 2015; He et al., 2020).

The spike wave index (SWI) of the non-rapid eye movement (NREM) phase was defined to quantify the epileptiform activity in the EEG during sleep. Accurate SWI quantification has important implications for clinical diagnosis and prognosis. Previously, SWI ≥ 85% was considered to be an important risk factor for poor prognosis of RE patients, mainly including cognitive impairment, frequent seizures and poor drug response (Tovia et al., 2011). Moreover, SWI = 50% was considered to be a cut-off value reflecting a good prognosis of patients, and patients with SWI < 50% were often considered to be at low risk (Parisi et al., 2017). Studies on SWI in the 50–85% range are still lacking, and its clinical significance has not been clearly defined. However, recent studies demonstrated that even RE patients with SWI between 50 and 85% suffered from failure of monotherapy and a poor long-term prognosis of cognitive function (Oztoprak et al., 2021; Ucar et al., 2022). Recently, it has been noted that RE patients with SWI ≥ 50% perform significantly worse on arithmetic calculations, executive functions, attention and memory tests than children with SWI < 50% (Zhang J. et al., 2020). Therefore, it is clinically significant to focus on RE patients whose SWI is within the 50–85% range. Due to long EEG examination time, even if a 24-h video EEG (VEEG) is taken, there is still a possibility of false negatives. Hence, multiple examinations are often required to determine the true SWI of patients during the NREM phase (Parisi et al., 2017). There is still a lack of reliable diagnostic markers that could confirm the diagnosis of these patients as early as possible.

Based on the fact that epilepsy is a network disorder (Royer et al., 2022), research on functional connectivity (FC) networks of RE patients is currently a hot topic. The default mode network (DMN) is a key network that is associated with cognitive function, mental behavior, and conscious arousal in reports that examine resting-state brain FC networks (Hsiao et al., 2013, 2015; Chen et al., 2018, 2019). The DMN-related regions consist of several cortical centers, including the posterior cingulate cortex (PCC), precuneus (PCu), lateral temporal cortex (LTC), medial temporal cortex (MTC), medial frontal cortex (MFC), and inferior parietal cortex (IPC) (Greicius et al., 2003; Buckner et al., 2008). An earlier study showed that RE patients not only had significantly reduced DMN activation during resting state, but also had less inactivation during cognitive work (Oser et al., 2014). A recent study suggested that interictal epileptiform discharges may disrupt the DMN in RE patients and may be responsible for cognitive impairment in RE patients (Zhang T. et al., 2020). Therefore, DMN is the most important observation target in resting-state imaging studies. Not only that, many studies using magnetoencephalography (MEG) to analyze the FC network revealed that the FC network can indicate the origin of epileptogenic foci and predict the prognosis of epilepsy patients (Nissen et al., 2017; van Mierlo et al., 2019). Due to a lack of FC network studies on SWI elevation during the NREM phase, determining whether there are objective DMN changes in this group of patients is of great importance.

With the rapid development of imaging technology, an increasing number of non-invasive examinations of the brain are being applied to study FC networks, such as, functional magnetic resonance imaging (fMRI), EEG and others (Danhofer et al., 2018; Chen et al., 2019). MEG is a novel detection method that has high temporal and spatial resolution. The signal of the MEG can pass through the cerebrospinal fluid, skull and other subcutaneous tissues without being attenuated, with less artifact interference (Barkley and Baumgartner, 2003; Baillet, 2017). Importantly, it is capable of exploring signals in the higher frequency bands of the human brain (Jacobs et al., 2012). But it is still worth mentioning that MEG is more affected by head motion than EEG, and that the amplitude of MEG signal is greatly affected by source distance, resulting in worse sensitivity to deep sources than EEG (Petrov and Sridhar, 2013). Also, it is less sensitive to radial currents (Hillebrand and Barnes, 2002; Baillet, 2017). Although the advantages and disadvantages of MEG are very obvious at present, with the development of software analysis methods and the progress of MEG detection, MEG is now considered by a large number of scholars to have a high potential in brain activity research.

In summary, based on this background, the present study has three goals. First, at the regional level, we aimed to explore the changes in spectral power in DMN-related regions at the resting-state in RE patients with mild SWI (50–85%) and typical RE patients with SWI < 50% compared to healthy controls (HC). At the FC level, we set out to investigate whether patients with mild SWI and typical RE patients demonstrate abnormal connectivity between DMN nodes in specific frequency bands. Finally, we aimed to evaluate whether objective DMN-based alterations may serve as a good neuroimaging biomarker to distinguish individuals with mild SWI from those with typical RE.

## Experimental procedures

### Subjects

A total of 40 patients aged 6–13 years with a diagnosis of RE that attended the outpatient clinics of the Department of Neurology of The Affiliated Brain Hospital of Nanjing Medical University and The Affiliated Children’s Hospital of Nanjing Medical University in China were recruited for this study. All enrolled RE patients had not taken ASMs prior to enrollment. All patients met the International League Against Epilepsy (ILAE) 2017 seizure classification criteria (Fisher et al., 2017). Furthermore, 38 of them met the following inclusion criteria. Among the cohort, there were 20 patients in the mild SWI group and 18 patients in the SWI < 50% group (typical RE patients). Meanwhile, we recruited 20 age- and sex-matched healthy children as controls in the society.

The inclusion criteria were as follows. (a) Patients who met the ILAE 2017 classification of epilepsy syndromes and were diagnosed with RE. (b) Patients who had undergone VEEG examinations of > 8 h three times or more, which showed high amplitude spikes that originate in the central temporal region with normal background waves. NREM phase SWI was calculated by dividing the spike-wave release time during NREM sleep phase by the total NREM sleep time. Patients in the NREM phase SWI in the range of 50–85% were defined as the mild SWI group, and patients with SWI < 50% were defined as the typical RE group. (c) Patients aged 6–13 years with no prior treatment for ASMs. (d) No other neurological or psychiatric disorders and negative MRI scanning results. (e) Patients and their legal guardians were willing to sign informed consent form, as required. The exclusion criteria were as follows. (a) Patients with metal implants in the body (i.e., vagus nerve stimulation devices, pacemakers, etc.). (b) Patients with a history of other neurological or psychiatric disorders, including traumatic brain injury and schizophrenia. (c) Patients with significant growth retardation.

All patients and their legal guardians were informed and agreed to the study protocol. All participants signed the informed consent form. The study protocol was granted approval by the Medical Ethics Committee of The Affiliated Brain Hospital of Nanjing Medical University and The Affiliated Children’s Hospital of Nanjing Medical University in China. Patients completed all tests required for enrollment on the day they received their diagnosis, without affecting their subsequent medication. All patients were seizure-free in the 72 h before and 48 h after the scan.

### Neuropsychological assessments

Based on prior studies (Yang et al., 2013), the Chinese version of the Wechsler Intelligence Scale for Children, Fourth Edition (WISC-IV) was utilized to investigate the intelligence level of all RE patients (Wechsler, 2007). The scale contains 10 core subtests and five additional subtests. The scale scores consist of Verbal Comprehension Index (VCI), Perceptual Reasoning Index (PRI), Working Memory Index (WMI), Processing Speed Index (PSI), and Full-scale Intelligence Quotient (FSIQ). The score of each sub-test of the scale is obtained using standard norm conversion, thus making the scores of subjects of different ages comparable (Wechsler, 2007). The scale has shown good reliability in previous studies (Baron, 2005). All tests of the WISC-IV were performed by clinicians specializing in child neuropsychology.

### Magnetoencephalography recordings

A whole-scalp CTF 275-channel MEG system (VSM MedTech Systems, Inc., Coquilam, BC, Canada) was utilized to record the activity of neuromagnetic signals. Prior to data acquisition, we removed all metal objects from each subject’s body. Three coils were placed and fixed at the root of the nose, as well as in front of the ears, to serve as anatomical markers for precise fusion with subsequent MRI. In order to capture sensor and background noise, we conducted a 3-min empty-room recording prior to recording the MEG data, which was used to calculate noise covariance for source analysis. The MEG data was recorded for six sets of 120 consecutive seconds at a sampling rate of 6,000 Hz. At the same time, electrooculography (EOG) and electrocardiography (ECG) recordings were carried out on each subject during the MEG recordings. During the data recording, each subject was asked to remain still and then relaxed in a supine position, gently closing their eyes, but avoiding falling asleep. Head position was determined before and after each data collection in order to ensure that the subject’s head movement error was limited to 5 mm or less. If a subject moved their head excessively or fell asleep during recording, then the set of data was discarded and re-recorded.

MRI scans of each subject were conducted using 3.0T-MRI (Siemens, Germany). The coordinates of MRI acquisition were calibrated by previously placed marker positions of the three coils in the MEG recordings in order to avoid MRI bias due to changes in head orientation. In this way, the anatomical position of the MRI can be determined after visualization of the MEG data, thereby ensuring that the MRI and MEG data of subjects may be accurately fused.

### Data preprocessing

In order to eliminate signals of non-brain activity and environmental artifacts from the MEG data, the following strategies were used. (1) All data were visually inspected, and where there were significant head position bias or artifactual segments due to noise interference, we removed the contaminated segments. (2) Power line contamination was removed through the use of a notch filter (50 Hz and its harmonics). (3) MEG data recording started after 3 min of empty-room recording in order to collect background and sensor noise, and noise covariance was calculated from offline source analysis. (4) Heartbeat and blink events identified from ECG and EOG data were utilized to define projectors independently using principal component analysis. Principal components meeting the artifact sensor topology were manually selected and excluded using orthogonal projection (Florin and Baillet, 2015). In addition, T1-weighted structural volume images were automatically reconstructed in the surface model through the use of a FreeSurfer image analysis package for source investigation.^1^ Topographical 3D descriptions of the brain surface generated using integrated geometric reconstructions of the scalp, brain gray matter and brain white matter was utilized to estimate the boundaries of gray and white matter. Next, a continuous segment of 60 s without spike discharges was chosen for each group of patients in order to avoid interference of the EEG signal by spike discharges. Finally, we chose the following frequency bands for MEG data analysis, including delta (2–4 Hz), theta (5–7 Hz), alpha (8–12 Hz), beta (15–29 Hz), gamma1 (30–59 Hz), and gamma2 (60–90 Hz).

### Spectral power

Depth-weighted minimum norm estimation (MNE) was utilized to estimate the source-level-based cortical activation from MEG data. A number of prior studies have demonstrated the robustness of the MNE method (Kanamori et al., 2013; Hincapié et al., 2016). A forward model of the MNE analysis was built through the use of an overlapping sphere method, which described each cortical vertex as a current dipole and included approximately 15,000 vertices. Next, an inverse operator that estimated the current source distribution of the sensor recording data was calculated as follows. (1) The source direction was constrained to be perpendicular to the cortical surface. (2) A depth-weighted algorithm was utilized to compensate for biases affecting the superficial source calculation. (3) The regularization value λ2 = 0.33 was used to reduce the numerical instability, reduce noise sensitivity of the MNE, and create a spatially smooth solution. The regularization parameter determines the weight of the MEG signal model relative to the background noise model, which is defined as the inverse of the signal-to-noise ratio (SNR) of the MEG record. The default SNR in Brainstorm software is “3,” which adopts the definition of SNR in the original MNE software (Gramfort et al., 2014). Brainstorm is a published software that is available for free online download under the GNU General Public License for conducting depth-weighted MNE studies.^2^

We chose 12 DMN-related brain regions as regions-of-interest (ROIs) using the Desikan-Killiany atlas, which includes bilateral PCC, PCu, LTC, MTC, MFC, and IPC. Specific Montreal Neurological Institute (MNI) coordinates were listed in Table 1. Cortical maps reflecting spectral power and FC for each subject were shown on the same source space: cortex from MNI152 anatomy. ROIs were selected from the default anatomical cortical surface (MNI152) according to an automatic anatomical marker template (Tzourio-Mazoyer et al., 2002). In order to estimate the source-dependent oscillatory power, the relative current powers of all vertices in the ROI were calculated. The power spectral density (PSD) of each ROI was calculated through the use of the Welch technique (5 s window duration; 50% overlap) (Niso et al., 2019; Tadel et al., 2019). The PSD values represented the spectral power of each individual. At each frequency band, the spectral power values were scaled in proportion to the total power over the entire spectrum.

**TABLE 1:** MNI coordinates (x, y, z) of the center of each DMN-related brain regions.

	Left cerebral hemisphere (mm)	Right cerebral hemisphere (mm)
Inferior parietal cortex	(−43.11, −47.02, 45.42)	(46.29, −47.59, 48.19)
Medial frontal cortex	(−5.44, 52.52, −8.86)	(7.83, 50.41, −8.52)
Medial temporal cortex	(−21.49, −17.29, −21.92)	(25.15, −16.3, −21.74)
Precuneus	(−7.59, −57.32, 46.64)	(9.69, −57.31, 42.36)
Posterior cingulate cortex	(−5.21, −44.17, 23.28)	(7.18, −43.09, 20.47)
Lateral temporal cortex	(−55.87, −34.98, −3.58)	(57.16, −38.57, −2.78)

MNI, montreal neurological institute; DMN, default mode network.


$$\mathrm{Relative}\,\mathrm{PSD}\,\left(f\right)=\mathrm{PSD}\,\left(f\right)/\sum_{i}\left[\mathrm{Total}\,\mathrm{PSD}\,\left({\text{f}}_{i}\right)\right]$$

Where f*_i_*’s are the individual frequency bands from the original PSD. The numerator of the formula represents the original PSD value of the current frequency band, and the denominator of the formula represents the sum of the original PSD values of all selected frequency bands. The relative PSD value is between 0 and 1, indicating the contribution of the current frequency band to the total signal power (Niso et al., 2019). Spectral power was reported to be normalized across brain regions and participants through the use of this method, allowing for better comparability across individuals (Niso et al., 2019).

### Functional connectivity analysis of resting-state data

Corrected amplitude envelope correlation (AEC-c) analysis was utilized to estimate oscillatory functional connectivity in the aforementioned DMN-related brain regions. Previous studies have reported that AEC-c analysis indicates strong repeatability and stability in the FC network research (Colclough et al., 2016). According to the method reported in the previous study, we orthogonalized the signal pairs before envelope computation to eliminate spurious connections due to volume conduction effects and field spread (Colclough et al., 2015). The amplitude envelope was defined as the absolute value of the Hilbert transform of a certain cortical oscillation, obtained from band-pass filtered cortical source activity for each frequency band. This reflects the fluctuation of amplitude over time (Brookes et al., 2011). If s(t) was an arbitrary time series, then the Hilbert transform is defined as:


$$s_{H}\,\left(t\right)=\frac{1}{\pi }\,\int_{-\infty }^{+\infty }\frac{s\,\left(\tau \right)}{\left(t-\tau \right)}\,d\,\tau$$

The Hilbert envelope was then divided into an n equal-length time periods, and the average value of the envelope in each time window was calculated. The Pearson correlations between these average envelopes were calculated as a measure of FC, which is reflected by the AEC-c value. The AEC-c values were calculated by correlating the amplitude envelopes of cortical oscillatory activity from the two ROIs. A high AEC-c value indicates that the amplitude envelope has strong synchronized fluctuations between the two cortical regions, reflecting a presence of strong FC between the two regions (Cheng et al., 2020; Godfrey and Singh, 2021). Finally, AEC-c values for all subjects under all ROIs were calculated, and the full 12 × 12 adjacency matrix was estimated.

### Statistical analysis

The demographic and clinical information was compared between the mild SWI group and SWI < 50% group through the use of independent samples *t*-test or chi-square test, as appropriate. The Kolmogorov-Smirnov test was used to assess the data distribution. The Kruskal-Wallis test was then used to compare group differences of spectral power for each ROI in each frequency band for each of the three groups of subjects, as well as group differences in AEC-c values that represent the FC strength between ROI nodes. We utilized *p* < 0.05 as the threshold for statistical significance, which was corrected using Bonferroni multiple comparison correction. Specifically, at the regional level (spectral power), there were 12 ROIs and 3 groups of subjects, so the *p*-value in the spectral power analysis needed to be corrected 12*3 = 36 times. At the FC level, we performed matrix analysis of 12 ROIs, with a total of 3 groups of subjects, so the *p*-value in the FC analysis needed to be corrected 66*3 = 198 times. In addition, Kruskal-Wallis tests suggested differences between the three groups, spectral power and AEC-c values with significant differences between the mild SWI and SWI < 50% groups were also determined using binary logistic regression analysis to determine the diagnostic value of the mild SWI group through the use of receiver operator characteristic (ROC) curve analysis, and area under curve (AUC) was plotted to investigate the diagnostic performance. All data are expressed as mean ± standard deviation (SD). All statistical analyses were performed using SPSS 24.0 (SPSS Inc., Chicago, IL, United States).

## Results

### Clinical information

Data from 38 RE patients and 20 HCs were included in this study. The patient group included 20 patients with NREM phase 50% ≤ SWI < 85% (mild SWI group), as well as 18 patients with typical RE (SWI < 50% group). The age of children in the HC group was 8.20 ± 1.58 years while the gender ratio male/female was 11:9, with age and gender ratio matching those of the other two groups. The detailed clinical data of mild SWI group and SWI < 50% groups are shown in Table 2. There were no significant differences with regards to the two groups in terms of gender distribution, age, course of disease, and number of seizures. Meanwhile, WISC-IV scores indicated no significant difference with regards to cognitive function between the mild SWI and SWI < 50% groups in the early stages of the disease course.

**TABLE 2:** Patients’ clinical data, mean ± SD.

Clinical characteristics	Mild SWI group (*n* = 20)	SWI < 50% group (*n* = 18)	*P*-*value*
Gender			0.78
Female	8	8	
Male	12	10	
Age at seizure onset, years	8.10 ± 1.57	8.01 ± 1.71	0.93
Age at scan, years	8.87 ± 2.64	8.79 ± 1.72	0.73
The course of epilepsy, months	0.96 ± 0.35	0.87 ± 0.32	0.82
The number of seizures	2.35 ± 0.75	2.28 ± 0.83	0.78
**WISC-IV scores**			
FSIQ	93.15 ± 9.12	98.50 ± 12.47	0.14
VCI	92.90 ± 10.11	98.94 ± 12.34	0.11
PRI	92.30 ± 14.09	95.83 ± 14.24	0.45
WMI	99.65 ± 8.76	100.11 ± 11.52	0.89
PSI	92.75 ± 10.00	97.44 ± 13.80	0.23

SWI, spike-wave index; NREM, non-rapid eye movement; WISC-IV, Wechsler Intelligence Scale for Children, Fourth Edition; FSIQ, full scale intelligence quotient; VCI, verbal comprehension index; PRI, perceptual reasoning index; WMI, working memory index; PSI, processing speed index.

### Spectral power in the default mode network

Interestingly, some DMN-related regions in both the mild SWI group and SWI < 50% group demonstrated frequency-dependent changes that were different from those in the HC group. Importantly, patients in the mild SWI group exhibited spectral power changes in specific frequency bands that were different from those in the SWI < 50% and HC groups early on in the course of the disease. The differences in the spectral power of the DMN brain regions among the three groups were largely concentrated in the delta and alpha bands. There were no significant differences in the spectral power of theta, beta, and gamma frequency bands.

#### Delta band (2–4 Hz)

**FIGURE 1:**
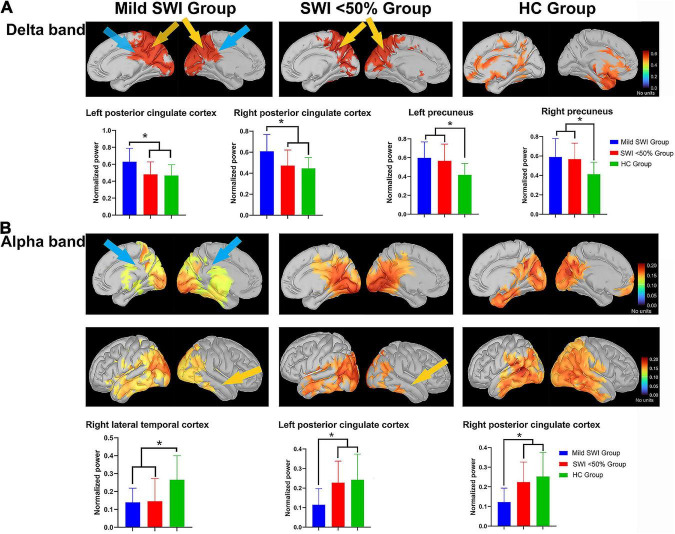
The significant differences regarding spectral power between the mild SWI group, SWI < 50% group and HC group. The blue arrows represent that the mild SWI group is significantly different from the other two groups, and the yellow arrows represent that both the mild SWI group and the SWI < 50% group were significantly different from the HC group. In the delta band **(A)**, the mild SWI group had significantly enhanced spectral power within the bilateral PCCs, and both the mild SWI group and SWI < 50% group had significantly enhanced spectral power in the bilateral PCu. In the alpha band **(B)**, the spectral power of the right LTC was significantly attenuated in both the mild SWI group and SWI < 50% group, and the spectral power of the bilateral PCCs in the mild SWI group was significantly attenuated. *P-values after correction were less than 0.05.

Across the three groups of subjects, there was a significant group difference in the spectral power of the left PCC (*p* = 0.002). Further comparisons revealed that the mild SWI group had significantly higher spectral power in the left PCC compared to the SWI < 50% (*p* = 0.005) and HC groups (*p* = 0.002). On the other hand, there were no significant differences between the SWI < 50% and HC groups. The spectral power of the right PCC was found to be significantly different between groups (*p* = 0.002). Further comparisons revealed that the mild SWI group had significantly higher spectral power in the right PCC compared to the SWI < 50% (*p* = 0.007) and HC groups (*p* = 0.001), while there was no significant difference seen between the SWI < 50% and HC groups. The spectral power of the left PCu was significantly different between groups (*p* = 0.002). Further comparison indicated that the spectral power in the mild SWI (*p* = 0.001) and SWI < 50% (*p* = 0.008) groups was significantly higher in the left PCu compared to the HC group, while there were no significant difference seen between the SWI < 50% and mild SWI groups. The spectral power of the right PCu was found to be significantly different between groups (*p* = 0.001). Further comparison demonstrated that the spectral power in the right PCu was significantly higher in the mild SWI (*p* = 0.006) and SWI < 50% (*p* = 0.001) groups compared to the HC group, while there were no significant difference seen between the SWI < 50% and mild SWI groups (Figure 1).

#### Alpha band (8–12 Hz)

Across the three different groups of subjects, there was a significant between-group difference in spectral power of the right LTC (*p* = 0.002). Further comparison demonstrated that the spectral power of the right LTC was significantly lower in the mild SWI (*p* = 0.004) and SWI < 50% (*p* = 0.002) groups compared to the HC group, while there was no significant difference between the SWI < 50% and mild SWI groups. There was found to be a significant intergroup difference in the spectral power of left PCC (*p* = 0.001). Further comparisons revealed that the spectral power of the left PCC in the mild SWI group was significantly lower compared to the SWI < 50% (*p* = 0.002) and HC groups (*p* = 0.001), while there was no significant difference between the SWI < 50% and HC groups. There was a significant group difference in the spectral power of the right PCC (*p* = 0.001). Further comparisons revealed that the spectral power of right PCC in the mild SWI group was significantly lower than compared to the SWI < 50% (*p* = 0.004) and HC groups (*p* = 0.001), while there was no significant difference between the SWI < 50% and HC groups (Figure 1). All the above *p*-values were obtained after Bonferroni correction.

In general, at the regional level, spectral power enhancement in delta band in bilateral PCC and attenuation in alpha band in bilateral PCC were characteristic changes in patients of mild SWI group. In addition, spectral power enhancement in the bilateral PCu in the delta band and attenuation in the right LTC in the alpha band were common in all RE patients.

### Functional connectivity in the default mode network

Herein, the FC of the DMN network was specifically altered across the different frequency bands. Compared to HC, the DMN network alterations in the mild SWI and SWI < 50% groups were mainly concentrated within the delta, theta, and alpha frequency bands. Additionally, there were no significant differences seen in the beta and gamma frequency bands in the subjects of the three groups.

#### Delta band (2–4 Hz)

**FIGURE 2:**
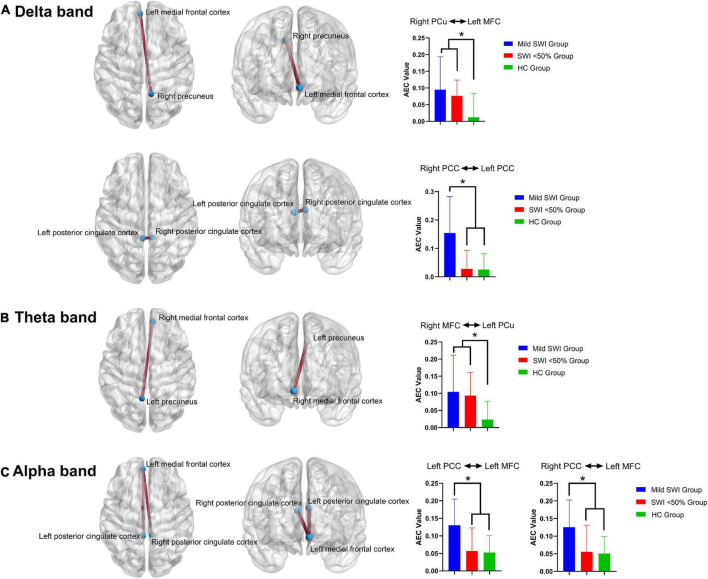
The significant differences regarding FC between the mild SWI group, SWI < 50% group and HC group. In the delta band **(A)**, the AEC-c value between the right PCu and left MFC were significantly increased in the mild SWI group and SWI < 50% group, while the AEC-c value between bilateral PCC in the mild SWI group was significantly increased. In the theta band **(B)**, the AEC-c value between the right MFC and left PCu were significantly increased in the mild SWI group and SWI < 50% group. In the alpha band **(C)**, the AEC-c value between the left PCC and the left MFC were significantly increased in the mild SWI group, and the AEC-c value between the right PCC and left MFC were significantly increased within the mild SWI group. *P-values after correction were less than 0.05.

Among the three groups, the AEC-c value between the right PCu and left MFC showed significant intergroup differences (*p* = 0.001). Further comparisons showed that the AEC-c value between right PCu and left MFC were significantly higher in the mild SWI group (*p* = 0.001) and SWI < 50% group (*p* = 0.002) compared to the HC group, while mild SWI and SWI < 50% groups did not show any significant differences. Among the three groups, the AEC-c value between the right PCC and left PCC demonstrated a significant difference (*p* = 0.001). Further comparisons showed that the AEC-c value between the bilateral PCC in the mild SWI group was significantly higher compared to the SWI < 50% group (*p* = 0.002) and HC group (*p* = 0.003). However, there was no significant difference between the HC and SWI < 50% group (Figure 2).

#### Theta band (5–7 Hz)

Among the three groups, the AEC-c values between the right MFC and left PCu showed significant differences between the groups (*p* = 0.001). Additionally, further comparison indicated that the AEC-c value between the right MFC and left PCu were significantly higher in the mild SWI (*p* = 0.002) and SWI < 50% (*p* = 0.002) groups compared to the HC group, while the mild SWI and SWI < 50% groups did not show any significant differences (Figure 2).

#### Alpha band (8–12 Hz)

Among the three groups, the AEC-c value between the left PCC and left MFC showed significant differences (*p* = 0.002). Further comparison demonstrated that the AEC-c value between the left PCC and left MFC were significantly higher in the mild SWI group compared to the SWI < 50% (*p* = 0.002) and HC (*p* = 0.002) groups. However, there was no significant difference between the HC and SWI < 50% groups. Among the three groups, the AEC-c value between the right PCC and left MFC indicated that there was a significant difference (*p* = 0.002). Further two-by-two comparisons indicated that the AEC-c value between the right PCC and left MFC were significantly higher in the mild SWI group compared to the SWI < 50% (*p* = 0.002) and HC (*p* = 0.002) groups. However, there were no significant differences between the HC and SWI < 50% groups (Figure 2). All the above *p*-values were obtained after Bonferroni correction.

In general, at the FC level, patients in the mild SWI group indicated increased AEC-c values between the bilateral PCC in the delta band and between the left MFC and bilateral PCC in the alpha band. Increased AEC-c values between the right PCu and left MFC in the delta band, and between the left PCu and right MFC in the theta band, were common across all RE patients.

### Logistic regression analysis

**FIGURE 3:**
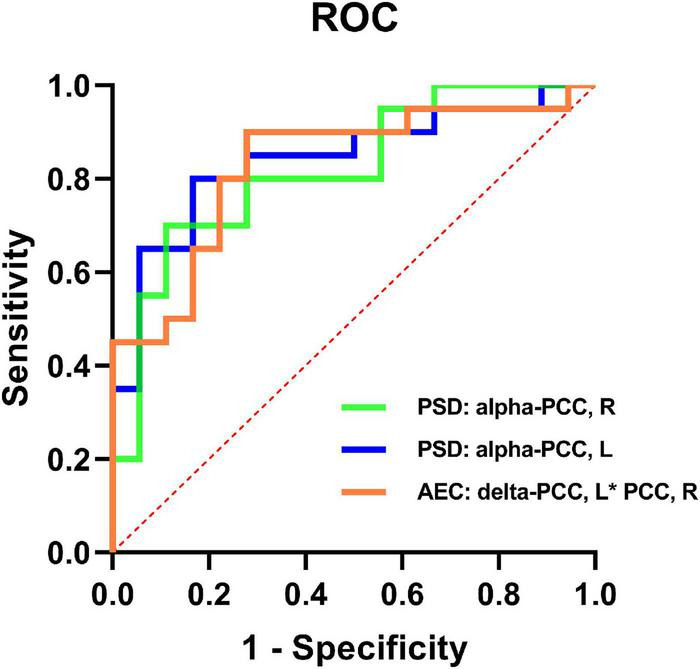
ROC curves of the spectral power of the bilateral PCCs in the alpha band and the AEC-c value between the bilateral PCCs in the delta band to discriminate mild SWI from typical RE individuals.

When the Kruskal-Wallis test confirmed that there were differences between the groups, binary logistic regression analysis was utilized to evaluate the AEC-c values and spectral power that had significant differences between the mild SWI group and the SWI < 50% group in order to identify predictors for mild SWI in early RE patients. Notably, by using mild SWI as the dependent variable, binary regression analysis indicated that the spectral power of the right PCC [95% CI (0.68, 0.95); *p* = 0.005] and the left PCC [95% CI (0.71, 0.97); *p* = 0.006] in alpha band and the AEC-c value between bilateral PCCs [95% CI (0.70, 0.96); *p* = 0.003] in delta band were statistically significant. The ROC curves for the three indicators are shown in Figure 3. The AUC area, sensitivity, specificity, and accuracy values of the three indicators are demonstrated in Table 3. In contrast, there was no statistical significance seen in the binary regression analysis when utilizing spectral power or AEC-c values from other DMN-related regions.

**TABLE 3:** Metrics for logistic regression models.

Indicators	AUC	Sensitivity	Specificity	Accuracy
Spectral power: alpha band- right PCC	0.84	0.75	0.72	0.71
Spectral power: alpha band- left PCC	0.82	0.80	0.83	0.71
AEC-c value: delta band- left PCC* right PCC	0.83	0.90	0.72	0.79

AUC, area under the curve; PCC, posterior cingulate cortex.

## Discussion

Herein, we explored objective DMN-related alterations among patients with mild SWI in the NREM phase with SWI in the 50–85% range, based on level of source spectral power activity and FC network connectivity within DMN brain regions, seeking imaging markers that may contribute to an early diagnosis. To the best of our knowledge, this is the first time that resting-state MEG has been utilized to explore alterations in the brain network patterns of early RE patients with mild SWI in the NREM phase. The present study contributed to the understanding of early changes within the brain network patterns in RE patients, and to the early differentiation between patients with typical RE and those with mild SWI.

Notably, the neurocognitive score (WISC-IV) did not demonstrate a statistically significant difference between the two groups of patients early on in the course of the disease. Even though these patients’ cognitive levels were still within the normal range, their cognitive scores, particularly those in the mild SWI group, were in general just above 90 on the FSIQ scale, which was at the bottom of the normal level of cognitive function (90–110 scores). Some reports have suggested that RE patients have some degree of cognitive impairment at an early stage (Yan et al., 2017; Li et al., 2020). However, there are still some reports that show that RE patients do not have any obvious early cognitive impairment, but show a certain degree of impairment in specific cognitive areas (i.e., memory, language, and reading) (Vannest et al., 2015; Wickens et al., 2017). The pathogenesis of the combined cognitive impairment in patients with RE is complex and there is still no conclusion. Currently, many scholars believe that frequent spike discharges during sleep are a more important cause of cognitive impairment among children with RE than seizures (Binnie, 2003; Vannest et al., 2016). The results of this current study do not support the presence of cognitive impairment in early RE patients. However, caution should be exercised regarding the possibility of combined cognitive impairment among early RE patients. The patients in this study had a short duration of disease, and the sample size remains small. Previous studies have demonstrated that cognitive impairment in RE patients gradually manifests itself as the disease progresses (Vannest et al., 2015). Therefore, this requires further confirmation in the future.

At the regional level, this present study systematically analyzed the changes that occur in various brain regions of the DMN in patients with NREM phase mild SWI and in RE patients at different frequency bands. The enhanced spectral power within the bilateral PCC region in the delta band and the diminished spectral power in the bilateral PCC region in the alpha band were characteristic of the patients within the mild SWI group. RE patients with mild SWI were previously easily ignored, but did have more frequent spike discharges, as well as a higher risk of cognitive impairment (Filippini et al., 2015; Parisi et al., 2017). It has been reported that while studying the effect of spike discharges on the whole brain power spectrum, changes were discovered in the activation pattern of DMN brain regions in RE patients in the resting-state, with abnormal results concentrated within the bilateral frontal lobe and PCC regions (Adebimpe et al., 2015). This study was the first to focus on the changes of magnetic source activity in the DMN brain regions of RE patients with mild SWI. Therefore, we speculate that the spike discharges within the interictal period, particularly in the NREM phase, lead to reorganization of DMN regions activation in the resting state in RE patients. Furthermore, the involvement of the PCC region is the most obvious in the early stages. Notably, patients with NREM phase mild SWI had an enhancement of spectral power in the delta band PCC region, although the spectral power was reduced within the alpha band PCC region. This suggested that the spectral power intensity of RE patients with mild SWI shifts from the alpha band to the delta band, and the spectral power in the alpha band tended to “slow down.” One study suggested that the PCC is considered to be a key node in the DMN-related region, as it is the only hub that interacts directly with additional DMN nodes (Hsiao et al., 2013). PCC is also often considered to be a key brain region that affects human cognitive function (Chen et al., 2018, 2019). Enhancement of the PCC region in the delta band has also been reported in patients with mild cognitive decline, and increased delta rhythm reflects deficits in brain activity or cognitive decline (Hsiao et al., 2013). As the cognitive function of patients in the mild SWI group remained at the bottom of the normal range, therefore, we speculate that the shift in spectral power intensity in bilateral PCC regions from the alpha band to the delta band may indicate underlying cognitive impairment in the ultra-early stage of the disease, although the cognitive impairment was not significant during this period. It remains to be confirmed by a longitudinal study with follow-up of this group of children, and further expansion of the sample size.

In parallel, FC enhancement between the bilateral PCC regions in the delta band and between the left MFC and bilateral PCC in the alpha band are characteristic alterations among patients in the mild SWI group. The maintenance of the normal state of the DMN network is important for regulating neurophysiological functions in humans (Lin et al., 2016). Additionally, many psychiatric and neurological disorders are thought to be related to abnormal activation of DMN brain regions, particularly within the PCC region (Maldjian et al., 2014; Burianová et al., 2017; Cheng et al., 2020). A previous study has shown that FC in the PCC region is often abnormally activated during the early stages of subjective cognitive decline, which suggests a compensatory mechanism before cognitive impairment (Hsiao et al., 2013). FC enhancement in the PCC region was considered to support a compensatory hypothesis that patients require additional neural network resources to maintain cognitive function (Zhang et al., 2009). As many studies have demonstrated some degree of cognitive impairment in RE patients, in the present study, patients with mild SWI have normal cognitive function, but they have a relatively short course of the disease. These patients had excessive FC between their bilateral PCCs. Hence, we speculate that they also had such a compensatory mechanism early on in the disease. The enhancement of FC between bilateral PCC regions early in the course of disease may be a compensatory mechanism for maintaining normal cognitive function in these patients. Further longitudinal follow-up studies with expanded sample sizes still need to be conducted to confirm this hypothesis. The DMN network can be separated into two parts at the functional level, with the anterior part focused on the MFC and the posterior part focused on the PCC (Buckner et al., 2008). The anterior-posterior connection, with the MFC and PCC as the main nodes, is thought to be highly relevant to conscious arousal, as well as cognitive function (Buckner and DiNicola, 2019). Herein, patients with mild SWI have enhanced FC in the delta and alpha frequency bands between the anterior and posterior brain regions of the DMN network, which was not found among patients with typical RE, suggesting that there is a disturbance in network topology among patients with mild SWI. We speculate that frequent spike discharges during the interictal period, particularly during the NREM period, cause patients’ DMN network to reorganize and produce excessive abnormal FC. Future longitudinal studies are still needed to dynamically observe the changes in FC among this group of patients.

To the best of our knowledge, the present study was the first to determine diagnostic values for NREM-phase mild SWI at both the magnetic source spectral power, as well as the FC network levels. The spectral power of the bilateral PCC and FC between them demonstrated good accuracy in differentiating mild SWI from typical RE, which suggests that PCC may also play a key role in the early identification of patients with mild SWI. As an intelligence quotient measure, FSIQ has been reported in previous study to be highly correlated with low frequency fluctuation of bilateral PCC and seizure frequency in RE patients (Tan et al., 2018). A similar recent study has demonstrated significant inactivation of the PCC region in RE patients with early cognitive impairment (Li et al., 2020). Thus, the PCC region has become a key brain region for research on RE, and alterations in the brain activity in the PCC region have the potential to reflect clinical features, as well as neuropsychological changes, in RE patients. The use of PCC spectral power and FC for early identification of patients with mild SWI will be valuable for future studies and will help clinicians develop more rational and individualized treatment plans at an early stage. Notably, at the spectral power level, bilateral PCus enhancement in the delta band, and right LTC attenuation in the alpha band are common features that occur in all RE patients. At the FC network level, the enhancement of the connection between the right PCu and the left MFC in the delta band and the enhancement between the left PCu and the right MFC in the theta band are common features of all RE patients. Although the sensitivity and specificity of these indicators for diagnosing RE were not explored in this study, once the high sensitivity and specificity of these indicators is validated in future studies, this could be utilized as a new imaging marker for an early RE diagnosis.

There are still some limitations to this study. Firstly, the sample size of this study is small. We developed strict criteria to include patients with typical RE without any other neuropsychological deficits and those with a SWI at 50–85% at the NREM stage. Hence, increased sample size is still needed in the future to validate the results of the current study. Secondly, the current study used the WISC-IV to score cognitive function of the patients, which, due to limitations of the WISC-IV itself, does not guarantee a systematic evaluation of an individual’s cognitive function within specific domains, such as language function. In the future, we will incorporate more scales to systematically evaluate neuropsychological changes within each individual. Thirdly, all current international MEG methods are not guaranteed to be completely free from artifacts and noise, although significant efforts have been made to eliminate the interference of artifacts and noise within the MEG signal. Further expansion of the sample size is needed in the future to validate the findings of this experiment. Fourth, the PCC region in this study may be a biomarker for early differentiation between typical RE and mild SWI patients, and no follow-up of this group of patients was conducted in this study. Therefore, longitudinal follow-up studies are needed in the future to dynamically observe the diagnostic validity of changes within this brain region. Finally, Since the aim of this study was to investigate DMN changes in RE patients with mild SWI and to detect early neuroimaging markers. Our study focused on finding possible diagnostic markers for RE patients with mild SWI. Therefore, we did not analyze the sensitivity and specificity of the results for all possible indicators of the diagnosis of RE patients. In future studies, we will further expand the sample size, analyze the sensitivity and specificity of all these indicators, and search for possible indicators of MEG diagnosis of RE.

## Conclusion

In conclusion, our study showed that the DMN brain regions of patients with mild SWI have characteristic alterations in spectral power as well as FC, unlike those of healthy children and typical RE patients. Moreover, typical RE patients and mild SWI patients have similar objective alterations at the FC and spectral power levels compared to healthy children. Furthermore, these alterations are band-specific. In addition, the spectral power of bilateral PCCs in the alpha band and the AEC-c values of bilateral PCCs in the delta band may be good indicators of early differentiation between mild SWI and typical RE.

## Data availability statement

The original contributions presented in this study are included in the article/supplementary material, further inquiries can be directed to the corresponding author.

## Ethics statement

The studies involving human participants were reviewed and approved by Medical Ethics Committee of The Affiliated Brain Hospital of Nanjing Medical University and the Affiliated Children’s Hospital of Nanjing Medical University in China. Written informed consent to participate in this study was provided by the participants or their legal guardian/next of kin.

## Author contributions

YL and YW designed the study. YL, YW, and PJ acquired the raw data. YL, YW, JS, and QC analyzed the data. YL wrote the manuscript. XW revised the manuscript. All authors read and approved the final submitted manuscript.

## Footnotes

1
https://surfer.nmr.mgh.harvard.edu

2
http://neuroimage.usc.edu/brainstorm
